# SIVsm Tat, Rev, and Nef1: functional characteristics of r-GV internalization on isotypes, cytokines, and intracellular degradation

**DOI:** 10.1186/1472-6750-10-54

**Published:** 2010-07-19

**Authors:** Marinko Sremac, Elizabeth S Stuart

**Affiliations:** 1 Department of Plant, Soil and Insect Sciences, University of Massachusetts, MA 01003, USA; 2 Department of Microbiology, University of Massachusetts, Amherst, MA 01003, USA

## Abstract

**Background:**

Recombinant gas vesicles (r-GV) from *Halobacterium sp*. strain SD109 expressing cassettes with different SIVsm inserts, have potential utility as an effective antigen display system for immunogen testing *in vivo *and for initial epitope assessments *in vitro*. Previous mouse model studies demonstrated immunization with r-GV expressing selected exogenous sequences elicited a prolonged immune response. Here we tested segments from three SIVsm genes (*tat, rev*, and *nef*) each surface displayed by r-GV. As with HIV, for SIVsm the proteins encoded by *tat, rev and nef *respectively serve critical and diverse functions: effects on efficient viral RNA polymerase II transcription, regulation of viral gene expression and effects on specific signaling functions through the assembly of multiprotein complexes. Humoral responses to r-GV^Tat, Rev or Nef1 ^elicited *in vivo*, associated changes in selected cell cytokine production following r-GV internalization, and the capacity of J774A.1 macrophage cells to degrade these internalized display/delivery particles *in vitro *were examined.

**Results:**

The *in vivo *studies involving r-GV immunizations and *in vitro *studies of r-GV uptake by J774A.1 macrophages demonstrated: (i) tests for antibody isotypes in immunized mice sera showed activation and re-stimulation of memory B cells, (ii) during long term immune response to the epitopes, primarily the IgG1 isotype was produced, (iii) *in vitro*, macrophage degradation of r-GV containing different SIVsm inserts occurred over a period of days resulting in an inherent slow breakdown and degradation of the SIVsm peptide inserts, (iv) vesicle specific GvpC, a larger protein, degraded more slowly than the recombinant peptide inserts and (v) *in vitro *uptake and degradation of the r-GV populations tested was associated with SIVsm insert specific patterns for cytokines IL-10, IL-12 and IL-18.

**Conclusions:**

Together these findings provide new information underscoring r-GV potential. They can clearly: display various exogenous peptides, be intracellularly degraded *in vitro *over a period of days, affect cell cytokine levels, and retain their self-adjuvanting capacity irrespective of the specific peptide expressed within the GvpC protein. These features support the cost effective generation of vaccine components, and provide a simple, self-adjuvanting system for assessing immune visibility of and specific responses to individual pathogen peptides.

## Background

Twenty eight years after the first cases were recognized, the HIV-1 pandemic continues to grow exponentially resulting in more than 42 million cases of individuals living with HIV worldwide. Constant virus replication in CD4 T lymphocytes initiates progressive immune defects and finally, after 6 to 10 years, results in acquired immunodeficiency syndrome (AIDS) and death. The course of the HIV infection has changed significantly with the development of new antiretroviral regimens that combine inhibitors of reverse transcription, virus protein cleavage, or even virus entry. They reduce viral burden and immune damage caused by HIV [1] but cannot fully eradicate the virus. Thus, lifelong therapy is expected to transform this otherwise lethal disease into a chronic, continuously treated infection by preventing the progression to AIDS. However, severe drug-related adverse effects and the development of drug resistance limit their efficacy, and the drugs have not been affordable for the vast majority of patients worldwide. Because a therapeutic breakthrough that would soon eradicate HIV or limit side effects appears unlikely at present, additional therapeutic strategies continue to be relevant to the lasting prevention of AIDS onset.

A better characterization of the initial host immune response to HIV-1 infection may help to define protective immunity to HIV-1. One such strategy might be to combine antiretroviral treatment with immune responses to HIV. Some immune control of HIV is evidenced by the temporal association of virus reduction and the emergence of HIV-specific T cells [2], however in the absence of a pre-infection stimulus, anti-HIV neutralizing antibodies normally develop too late to play a key role during natural infections. Findings have suggested that cellular immunity is involved in the initial control of virus replication in primary HIV-1 infection and indicate a role for CTL in protective immunity to HIV-1 *in vivo*. Importantly, analyses of vaccination studies in nonhuman primate have indicated that single viral epitope-specific CTL responses may not be sufficient to block infection with pathogenic SIV [3]. In turn this suggests that the generation of broader responses that target multiple viral epitopes may be critical to the development of effective protection against AIDS. Thus, a recent alternative approach has involved the use of multiple HIV antigens and the inclusion of both structural and regulatory antigens [4]. An indication that this approach can be more effective has recently been verified in that in animals, protection from heterologous SHIV challenge was observed only with immunization using an aggregated, multiantigen subunit protein vaccine that incorporated the structural protein Env and the regulatory proteins Tat and Nef [5,6]. When individually immunized using the same antigens, animals were not protected. Additionally, studies demonstrated improved protection from AIDS and CD4^+^T-cell depletion in animals immunized with a combination of antigens [7].

Inherently, the microparticulate gas vesicle organelles produced by *Halobacterium sp*. NRC-1 have numerous characteristics which would be desirable in an antigen presenting system and thus potentially can have importance for applications in vaccine development. The organelles are readily amenable to genetic engineering techniques that produce gas vesicles expressing on their surfaces the selected exogenous amino acid sequences, and the presence of surface borne peptide sequences has been assessed and verified *in vivo *[8]. Unlike most phage or animal virus-derived vaccine components, the gas vesicle structure does not contain nucleic acid. As a result, with the proteinaceous gas vesicle system the potential effects associated with co-introduction of DNA sequences, inherent in viral or phage based vaccines, can be completely avoided. The gas vesicles are readily, rapidly and inexpensively isolated from the bacteria by lysis in low salt and the isolated nanoparticles are uniquely stable. Large-scale production of gas vesicles is simple since these organelles float upon release from the *Halobacteria *and thus are easily harvested. Since they are non-toxic to animals and humans, gas vesicles should have utility for use as oral as well as parenteral delivery vehicles.

As initially demonstrated, gas vesicles from a mutant halophilic archaea SD 109, can be engineered to display various peptides by insertion of exogenous pathogen DNA sequences into a site engineered into the *gvpC *gene. Thus, expression system plasmids were generated in which SIVsm gene segments were inserted into site "*d*" engineered within *gvpC *gene [9]. The plasmid constructs were subsequently used to transform the Vac^- ^(minus) mutant, *Halobacterium *NRC-1 strain SD109 and the recombinant gas vesicles harvested and isolated as described in detail previously [8]. These nanoparticles were then pre-tested for immunogenicity, antigenicity and the activation of immunologic memory [9].

In the present study we examined sera from different times post-booster and post re-immunization to assess the antibody isotype elicited *in vivo *by populations of gas vesicles (r-GV^Tat, Rev and Nef1^) each expressing a selected SIVsm peptide sequence. Using an *in vitro *macrophage cell culture system we then tested for possible effects of gas vesicle uptake on cytokines produced by cultured macrophages through quantification of three (3) key examples: IL-10, IL-12 and IL-18. Finally, given the known, ultra stable nature of the gas vesicle nanoparticles [10,11] we used *in vitro *culture in conjunction with (i) immunofluorescence microscopy and (ii) western blot assessments to examine SIVsm and GvpC molecular weight species as indicators of their degradation. Thus, the changing intracellular distribution patterns of GvpC positive protein and the incorporated SIVsm peptide, as well as the molecular weight species of degradation products were specifically examined.

## Results

Immunoglobulins (Ig) are glycoproteins (antibodies) present in serum and tissue fluids that recognize and bind their cognate antigen. They constitute the adaptive, humoral immune responses, are non invasively accessible for testing and their utility *in vivo *is in part a function of their specific isotype. As a first step in the present studies, we therefore evaluated our archived serum to assess antibodies elicited by recombinant gas vesicles displaying an SIVsm peptide inserts (r-GV^Tat, Rev, and Nef1^) as a function of immunization status and overall elapsed time post-immunization or booster (see Table 1).

**Table 1:** Immunization regime of the BALB/c mice using r-GV.

Time points	C, Tat, Rev, Nef1
DOB	10/03/03

Prebleed	01/08/04

1^st ^immunization	01/08/04

2 weeks/immunization	01/22/04

4 weeks/immunization + 1^st ^boost	02/05/04

4 weeks/1^st ^boost + 2^nd ^boost	03/04/04

2 weeks/2^nd ^boost	03/18/04

4 weeks/2^nd ^boost	04/01/04

8 weeks/2^nd ^boost	04/29/04

12 weeks/2^nd ^boost	05/20/04

17 weeks/2^nd ^boost + re-immunization	06/24/04

10 days/re-immunization	07/04/04

Final bleed	04/15/05

### Isotyping of immune sera

**Figure 1:**
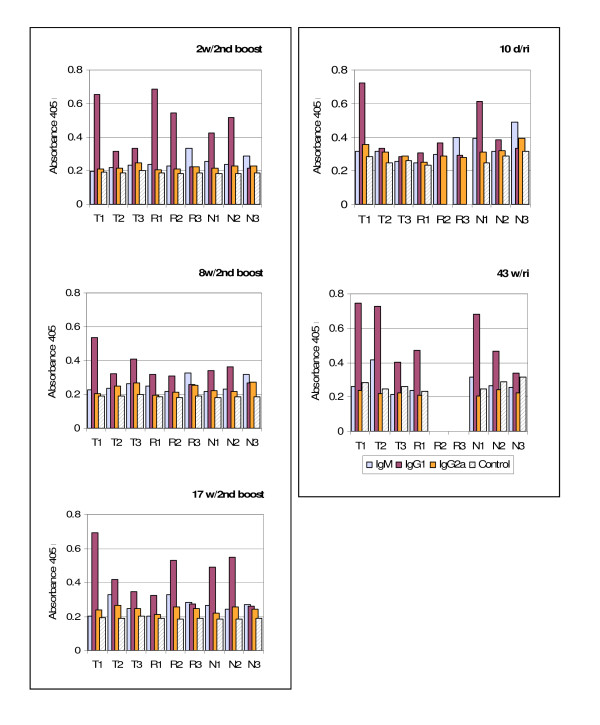
**Predominant Isotype Identification**. Isotypes of sera collected at different post booster or re-immunization times were determined using sera pre-adsorbed with wt-gas vesicles and assessed using isolated recombinant GV as the SIVsm antigen source. As detailed in the methods, sera diluted to the pre-established optimum were incubated in the r-GV pre-coated wells. Following rinses and incubation with pre-conjugated isotype specific antibody, isotype specific binding was detected by addition of pNPP substrate and absorbances quantified at 405 nm.

Based on results from the preliminary sera screenings, IgG1, IgG2a and IgM were selected for evaluation utilizing the SouthernBiotech isotyping kit as described in the methods. The assay results are presented graphically in Figure 1 which plots the isotypes detected at the optimum antigen specific antibody dilution. This antigen specific approach allowed determining the predominant isotype elicited and present at different times post-immunizaton or booster.

As the histograms in panels of Figure 1 show, with two exceptions (R3 and N3), IgG1 is the predominant isotype at 2, 8 and 17 weeks post second booster. Seventeen weeks later, the mice were re-immunized using aliquots of the same r-GV preparations and sera collected at 10 days and again at 43 weeks post re-immunization. The isotyping results for samples from these time points, shown in Figure 1, confirm that for all the mice tested, IgG1 remains the predominant isotype including the assessments of sera drawn at 43 weeks post re-immunization. For sera of all animals, at the optimum antigen specific dilution, IgG1 remained the predominant isotype and its signal was greater than that of the 10 day post re-immunization samples indicating the specific antibody responses continued to develop over time.

### Determination of key cytokines elicited from J774A.1 macrophage cells

In recent years the expression "cytokine network" has become commonly accepted in biomedical research and that network now includes several hundred members. Using our *in vitro *monolayer system this study assessed the levels of three key cytokines as a function of r-GV uptake by macrophage cell monolayers. The studies had two aims: first to evaluate whether uptake of gas vesicle particles by these monolayer cells could influence the detectible levels of cytokines in the surrounding media and second, to determine whether there were differential cytokine levels as a correlate of the specific SIVsm peptide expressed by the r-GVs applied to the individual monolayer cells. Positive findings would add to a profile that characterizes peptide specific effects *in vitro *as well as to the potential utility of this nanoparticle delivery system.

There are known interactive influences for each cytokine and macrophages normally produce IL-10, IL-12 and, IL-18, among others. IL-12 and IL-18 have important roles in inducing TH1 responses while IL-10 can antagonize the generation of TH1 subset of helper T cells and enhance proliferation of B cells. Further, macrophages are widely distributed in mammalian tissues and fluids. Therefore assessment utilized monolayers of J774A.1 murine macrophages and the protocols for *in vitro *r-GV application, followed by quantification of cytokines in the culture media as described in the methods section. To test whether there might be r-GV insert specific differences, cell monolayers were incubated for 2.5-3 h with wt-GV (NRC-1) a control, or with the different r-GV^Tat, Rev or Nef1^. At the times specified after GV removal and rinsing, aliquots of culture media were harvested and stored at -20°C until assessed for their content of the selected cytokines. Cytokine quantifications used point to point curves generated by assay of the individual purified cytokines provided in the cytokine kits and the calculation methods recommended by the kit manufacturer. Concentrations of the three different cytokines were assayed in media of cultured cells receiving no gas vesicles, media from duplicate cultures treated with one of the different SIVsm insert containing r-GV populations, or with the insert negative wild type control from NRC-1. Table 2 presents these results which are snapshots in time of individual cytokine levels with the elevated levels shown in bold and shows there are notable differences in concentrations, and these vary as a function of the SIVsm insert and also the time post application. Thus for r-GV^Tat or Rev^samples, concentrations of IL-10 and IL-12 were maximal at 12 h post application (185.311 and 147.218 pg/ml respectively), then declined. For samples of r-GV^Nef1^, the peak IL-10 concentration was much higher and occurred at 24 h (537.38 pg/ml); although lower in the 48 h samples, it was still elevated (168.622 pg/ml). For IL-12, maximal concentration again occurred early at 12 h for r-GV^Tat ^treated cultures (52.680 pg/ml), then decreased to the level of the insert negative NRC-1 in the 48 h sample. In contrast, for r-GV^Rev or Nef1 ^media, increased pg/ml were detected only in the 48 h samples with Nef1 exhibiting the higher concentration (54.730 pg/ml). Finally for IL-18, samples from r-GV^Tat or Rev ^treated cultures, the 12 h samples displayed some elevation in concentration (85.332 and 99.325 pg/ml) that somewhat exceeds the elevated levels detected in samples from the cultures exposed to the insert negative control, gas vesicles from NRC-1 (72.51 pg/ml). At later times, IL-18 levels were additionally elevated by r-GV^Tat^, with the higher levels occurring respectively at 24 and 48 h. (115.166 and 106.121 pg/ml). In r-GV^Rev ^treated samples, the 12 h time displayed elevated IL-18 (99.325 pg/ml) but this decreased to ~half that concentration in the 24 h and 48 h post-addition samples. In contrast, at the different times tested, monolayers treated with r-GV^Nef1 ^displayed no significant increases in IL-18 concentrations (68.855-72.456 pg/ml). In fact the levels were marginally lower than those from samples from insert negative NRC-1 cultures (72.51-78.739 pg/ml). Therefore these assays indicate that unlike assays for r-GV^Tat and Rev^, r-GV^Nef1 ^elicited no significant insert specific increase in IL-18 concentrations.

**Table 2:** Assessment of the cytokine concentrations (pg/ml) at 12, 24 and 48 hours post gas vesicle addition.

	IL-10 (pg/ml)			IL-12 (pg/ml)			IL-18 (pg/ml)		
	
	12 h	24 h	48 h	12 h	24 h	48 h	12 h	24 h	48 h
**Media^†^**	29.791	32.794	31.677	7.276	10.908	13	17.037	15.163	10.606

**NRC-1^‡^**	27.037	28.712	28.774	11.795	9.643	13.086	72.51	76.747	78.739

**Tat**	**185.311**	28.476	31.404	**52.680**	14.048	13.653	85.332	**115.166**	106.121

**Rev**	**147.218**	67.513	37.792	9.959	10.693	**19.830**	**99.325**	56.274	55.549

**Nef1**	31.739	**537.384**	**168.622**	8.675	6.498	**54.730**	68.855	68.935	**72.456**

**^† ^**As detailed in the methods, macrophage monolayers received media only.

**^‡ ^**Control wells received the wild type gas vesicles NRC-1.

Although these data are "snapshots", nevertheless they indicate there are quantifiable, SIVsm insert specific differences and time dependent effects on cytokines produced by J774A.1 cell following the application and internalization of r-GV^Tat, Rev or Nef1^. Thus in the context of an antigen delivery vehicle, there clearly is a detectible, insert specific impact on cytokine production by recipient monolayers. These results therefore add to an overall picture characterizing the immunogen as delivered via the r-GV system. They also suggest r-GVs could potentially be further designed for a co-delivery specifically aimed at simultaneously affecting cytokine levels.

### Intracellular fate of gas vesicles

At 50-1000 nm, individual gas vesicle nanoparticles are below the limit of light microscope resolution [9] but clusters of them, and of their breakdown products are detectible by immunostaining. Gas vesicles are remarkably stable particles [10-12] and thus may inherently result in a slow release of the incorporated peptide sequences. Therefore r-GV, their fate and susceptibility to timely degradation constitute critical parameters in context of *in vivo *applications.

### Immuno-microscopy assessment of r-GV intracellular fate

**Figure 2:**
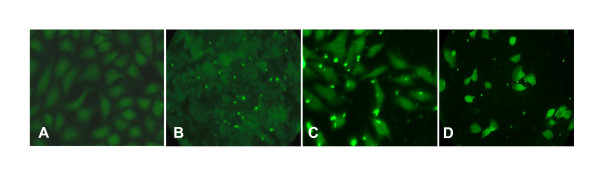
**Demonstration of gas vesicles phagocytosis by J774A.1 monolayer cells**. Gas vesicle preparations were added to monolayer cells as described, subsequently removed and the monolayers rinsed with fresh media, then maintained in culture. These representative 12 h post treatment micrographs of immunostained samples demonstrate that appropriate antibody detects each of the SIV encoded peptides in numerous cells of the r-GV treated monolayers. Control monolayers - **A**, received media only. Experimental monolayers received media containing purified gas vesicle preparations expressing Tat - **B**, Rev - **C **or Nef1 - **D**. Original magnification 400×.

Studies directed at examining the post-phagocytic fate of the recombinant nanoparticles used specific antibody probes to detect the SIVsm encoded peptides and the GvpC protein per se. This protein contains the insert and specifically identifies gas vesicle particles. Using the protocols specified in the methods, the J774A.1 macrophage cell line monolayers were prepared and incubated with r-GV then fixed as described at the different, specified times and stored at -20°C for subsequent assessments. Immunostaining with appropriate primary antibody allows immunofluorescent detection of r-GV delivered SIVsm peptides -Tat, Rev or Nef1 and separately, of the gas vesicle protein, GvpC. The initial distribution of r-GV material following internalization by monolayer cells is shown by the low magnification (400×) micrograph in Figure 2. It provides an overview of representative control and r-GV^Tat, Rev and Nef1 ^treated monolayers. These samples were harvested at 12 h post r-GV treatment and subsequently immunostained with the anti-SHIV monkey plasma which identifies the SIVsm peptide. Use of this plasma verifies that antibodies elicited during *in vivo *infection with live, native SHIV recognizes the r-GV^SIVsm ^peptides synthesized by the halobacteria as if they were native SIV protein. The monolayer in panel A of Figure 2 is a control to test for non specific antibody binding. It was not incubated with gas vesicle preparation and clearly the monolayer exhibits only a background fluorescence signal. In contrast, monolayers treated with r-GV show numerous cell associated fluorescence positive regions in Tat and Rev treated monolayers (Panel B and C). For the larger Nef1 insert (Panel D), there also are cells that appear to "glow". This fluorescence labelling shows the more extensive binding of anti-SHIV primary antibody to the larger Nef1 peptide contained in r-GV^Nef1 ^particles.

**Figure 3:**
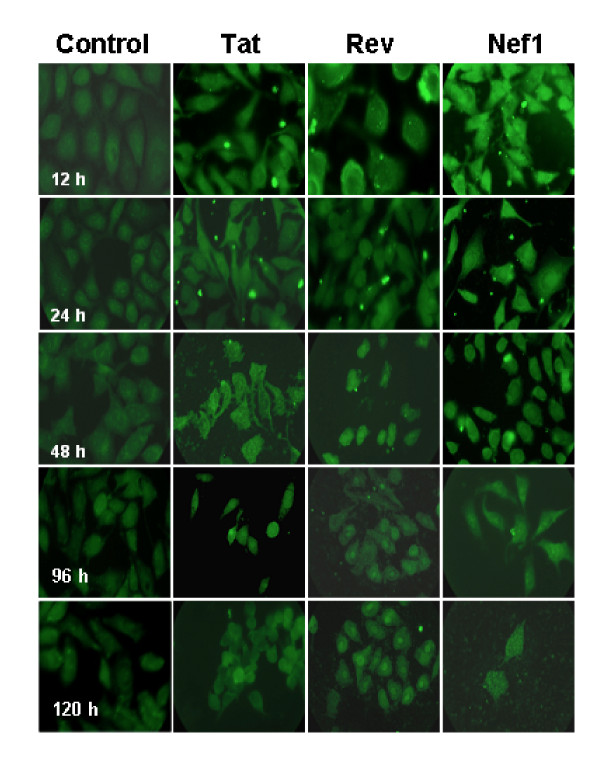
**Intracellular degradation of SIV specific peptides from gas vesicle treated monolayers**. Immunostaining of r-GV^Tat. Rev or Nef1 ^treated monolayers with anti-SHIV antisera demonstrates the time dependent disappearance of these epitopes from the macrophages. Note that for all three, the epitope specific immunostaining diminishes over time: 24-120 h post application. Additionally, the level of fluorescence detected is consistent with the SIV insert size. For the samples harvested at 48 h post addition, the larger Nef1 insert, expressed by r-GV^Nef1 ^appears more intensely stained than the inserts for r-GV^Tat ^or r-GV^Rev^. Original magnification 1000 ×.

A second, critical characteristic is the susceptibility of the r-GV delivery vehicle to intracellular degradation that releases the SIV inserts for processing by the cell. The fate of the SIVsm insert was followed in immunostained samples harvested over a period of 12-120 h and representative 1,000 × images of immounostain results are presented in Figure 3. As would be anticipated, these show that the SIVsm specific staining appears to become more diffusely distributed in the cells both as a function of the elapsed time, and the specific insert. At 12 h, SHIV positive particles (r-GV^Tat, Rev or Nef1^) appear to be associated with the monolayer cells, internalized by them, often as a cluster (ex. Rev, upper right) or internalized to produce a somewhat more diffuse green fluorescence (ex. Nef1). By 48 h the bright punctuate staining of Rev and Tat has diminished, becoming more diffuse, and these cells do not 'glow'. However for Nef1, the largest of the SIVsm encoded insert tested, distinct regions of 'glow' remain, likely because the degradation of this larger SIV insert remains less complete. However by 96 h, the immunostaining for Nef1 also has become more generalized. Interestingly, for the sample receiving r-GV^Rev^, nuclear regions of the cells immunostain quite brightly and in the 120 h Rev sample, this apparent nuclear association is clearly evident. Though not as distinct, a similar nuclear association occurs with the Tat insert. In contrast, the r-GV^Nef1 ^treated sample does not exhibit this distribution. Additionally, at the 120 h time, the diffuse, lightly stained areas detected in the cytoplasm of the r-GV^Nef1 ^sample appear similar to the cytoplasmic distributions detectible in the 48 h r-GV^Tat ^sample. This later appearance in the r-GV^Nef1 ^treated monolayer might be a function of slower breakdown of this larger expressed peptide. As expected, for each of the three different r-GV populations, the immunostaining of the incorporated SIVsm derived inserts is consistent with ongoing r-GV breakdown of the individual clusters and concomitant disappearance of detectible, brightly fluorescent particle clusters.

**Figure 4:**
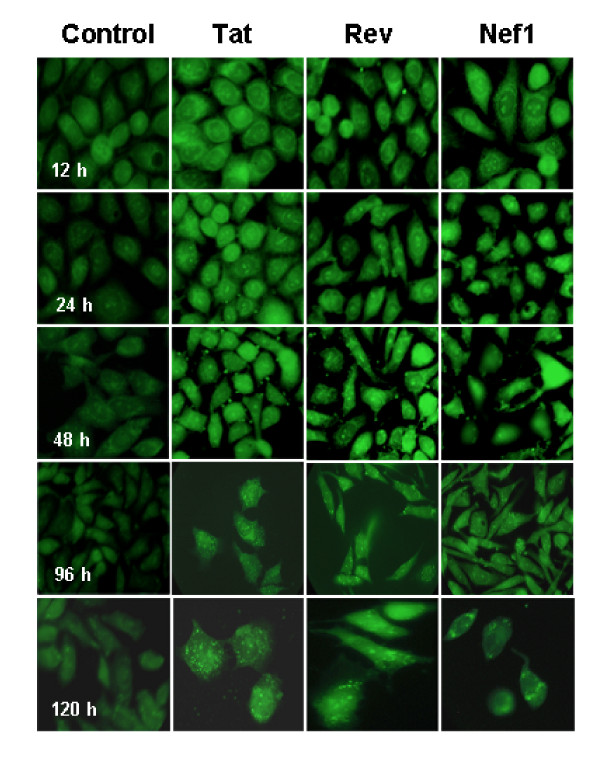
**Intracellular degradation of GvpC specific protein of gas vesicles**. Companion J774A.1 monolayers were prepared under conditions identical to those used for samples in figure 3 and similarly examined microscopically. This panel, immunostained with rabbit anti-GvpC antibody, demonstrates this gas vesicle specific protein also disappears over time. The quantities of GvpC protein remaining at the 120 h time appear greater than that observed for the SIVsm inserts. This would be consistent with the larger size of this protein and likely would support continued perturbation of cells following particle phagocytosis. Original magnification 1000 ×.

Other companion monolayers were similarly rinsed, fixed, then immunostained using anti-GvpC specific antibody to detect the larger, gas vesicle specific "C" protein and Figure 4 presents a temporal series of representative images from 12-120 h post r-GV application. Relative to the SIVsm inserts, GvpC is a large protein and like gas vesicle proteins in general, is resistant to dissociation/degradation [10,11]. Immunostaining to identify GvpC initially gives rise to cells exhibiting an overall glow that becomes significantly diminished by the 96 h time point and this would be consistent with a slow degradation of GvpC. However, positive but muted and somewhat diffuse punctuate immunostaining remains at the 120 h time and can support suggesting that full degradation of GvpC remains incomplete at this time.

In the context of a display/delivery particle, it is important that cells can both internalize the r-GVs, and successfully degrade them, allowing release of the pathogen specific insert they carry. The observed level and distribution of immuno-reactive GvpC material in monolayers at the 120 h time is fully consistent with ongoing particle degradation. Additionally, the decreasing brightness of the immunostaining supports suggesting the degradation time frame for and perturbation by GvpC will extend beyond the time required for the degradation of SIVsm inserts.

### Western blot assessments verify intracellular breakdown of SIVsm and GvpC components of r-GV

Ultimately, the processing and removal of immunogens and their carriers is a significant factor for *in vivo *applications. Therefore the fate of the GvpC and SIVsm components detected by immuno fluorescence microscopy during the late stages post r-GV application were examined further. Based on the patterns observed in these preparations, samples from the 96 h and 120 h were additionally assessed to verify protein breakdown. Other J774A.1 monolayers were incubated with the gas vesicle preparations under the same conditions, then rinsed and harvested. These samples were assessed by preparing cell lysates and separating the protein species using 4-12% SDS-PAGE gradient gels as detailed in the methods.

**Figure 5:**
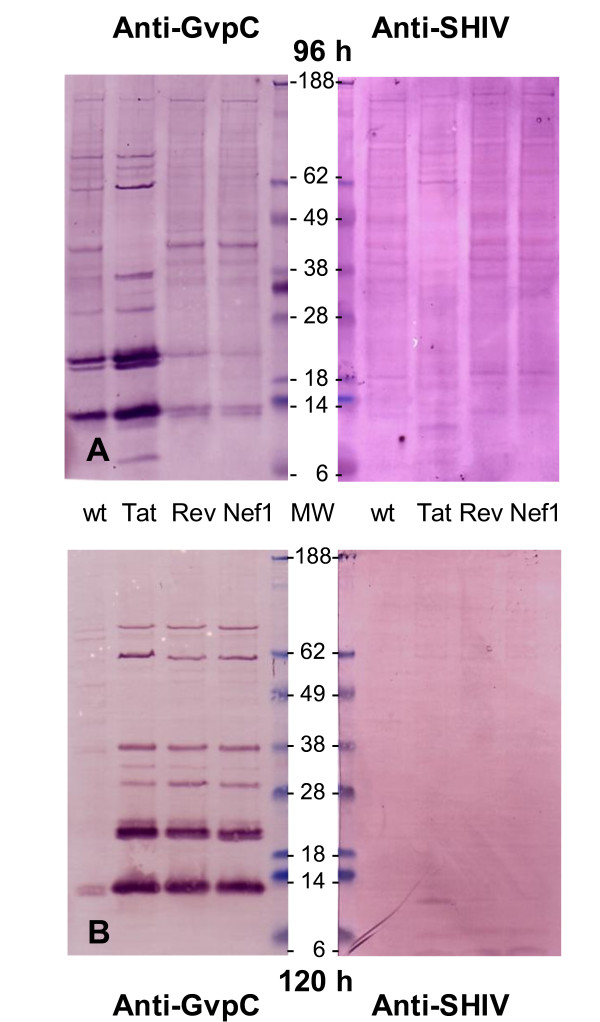
**Differential breakdown of GvpC and SIVsm components demonstrated by western blot**. Monolayer samples were harvested at different times post application of gas vesicles, then mixed with 5× SDS-PAGE sample buffer and subsequently assessed through western blot detection of GvpC or SIVsm material as described in the methods. These representative western blots demonstrate that at both the 96 and 120 h times examined, strong bands detecting GvpC are evident. For the much smaller SIV component of the same gas vesicles, numerous weak bands were evident at the 96 h time but by 120 h, the bands detected were very light and evident only at molecular weights ≤ 14 kDa.

In acrylamide gels, pure intact GvpC protein behaves anomalously relative to its actual molecular weight, migrating as a species of ~60 kDa [9]. Following separation using SDS-PAGE, and transfer to PVDF membrane, GvpC peptides and the expressed SIVsm insert were detected using the same anti-GvpC antibody stock and anti-SHIVsm monkey plasma utilized for the monolayers in immunostaining studies; companion halves of blot membranes were probed with the respective specific antibodies. Figure 5 presents typical Western blots of samples from the 96 h (upper blot pair) and 120 h (lower blot pair) time points, probed respectively with anti-GvpC antibody or anti-SHIVsm plasma. GvpC is synthesized by the recombinant bacteria and an integral, surface displayed component of these gas vesicles. The observed immunostained bands of differing kDa are a reflection of the molecular weights of GvpC breakdown products; other associated degradation resistant gas vesicle structural proteins also would be expected and if still integrated with GvpC, would affect its migration in gels [9]. The presence of multiple protein bands identified for r-GV samples and for the SHIV peptide is consistent with the immunostain microscopic observations which also suggested r-GV break down is a process that continues over a period of days. The membrane half probed with anti-GvpC antibody specifically identifies GvpC protein and peptides. It detected a number of bands at a variety of molecular weights, rather than the single ~60 kDa band that would characterize intact GvpC protein. The majority of bands in blots of the 96, and 120 h samples appear at < ~40 kDa with heavily stained bands detected at ~20 kDa and at somewhat at less than 14 kDa. The immunostain of these bands is notably stronger at the 120 h time, indicating GvpC breakdown is continuing and gives rise to these species, but clearly is still incomplete. Importantly, some bands may exhibit anomalous migration due to gas vesicle protein resistance to the standard protein dissociation protocols [10,11].

Slow disassembly and intracellular degradation would not be unexpected for gas vesicle proteins and likely perturbation of cells by partially degraded GvpC fragments would continue for an additional, but finite period of time. In contrast, the companion SIVsm inserts of Rev, Tat and Nef1 remain detectible but for the 96 h sample, the peptides immunostain lightly with the anti-SHIV antibody. Notably the inserts are an integral part of GvpC and like other GV proteins, GvpC is resistant to degradation. Multiple but light staining bands detected with the anti SHIV antibody would therefore be expected. At 120 h, the bands recognized by anti-SHIV antibody are minimally in evidence. No immunoreactive high molecular weight bands are obvious and the molecular weight species of ~6 kDa or less now are only minimally detectible. Clearly therefore, despite their integration into the original GvpC framework protein, the SIVsm inserts are accessible to degradative processing. Therefore these foreign proteins ultimately should be cleared by cells.

## Discussion

The studies presented above were designed to further assess practical aspects of the recombinant gas vesicle display/delivery system through *in vitro *and *in vivo *studies. As a self-adjuvanting, highly stable component [13], r-GV have significant potential as an alternative delivery system and thus the studies reported were designed to provide additional characterizations. The SIV fragments tested were selected because they are key immunogens examined as relevant gene products in other published research [14,15] Those studies had shown toxoids of Tat or Tat and Rev, or delivery of aggregated multiantigens, provided some disease attenuation [5,6] whereas use of Tat alone for example, provided only temporary infection containment. This was interpreted to mean a single antigen might not be adequate to provide full and/or long term protection [14-19]. Our recombinant system inherently supports the expression and delivery of various epitopes [8,9,13] and could be readily adapted if important, new peptides were identified. Thus combinations of r-GV particles could be easily created for use *in vivo *and this would provide exposure to multiple antigen fragments and thereby elicit a variety of peptide specific antibodies.

Different isotypes bind to cell surface expressed Fc receptors *in vivo *and the resulting receptor derived cell stimulation then will initiate a cascade of effects that can impact the local milieu, and ultimately cell functions. Utilizing the optimum antigen specific dilution of each antibody sample as determined by pre-titration using the specific Tat, Rev, and Nef1 antigens, the present studies identified the isotype(s) elicited by r-GV immunizations. These analyses showed IgG (primarily IgG1 with some instances of minimal IgG2a) was the predominant immunoglobulin species (Figure 1). Although for the R3 and N3 groups, IgM was predominant at 10 days, at 43 weeks, in the absence of any subsequent booster, IgG1 was the dominant isotype in all samples.

This can be relevant *in vivo *since antibody isotypes impact downstream events through antibody binding to cell displayed Fc receptors [20]. Additionally, the continued presence of antigen specific mouse antibodies 301 days (43 weeks) after the final re-immunization, is potentially an important feature for an immunizing agent and likely to be relevant in terms of practical utility. The isotyping data also inherently demonstrated that the peptides encoded by the expressed small DNA inserts (*tat*-150 bp and. *rev*-243 bp), are immunogenic and as shown in Figure 3, relative to the expressed *nef1 *encoded insert (642 bp) appear to be degraded more slowly than their actual size would predict. This finding may reflect the central location of these encoded small peptide inserts, 50 and 81 amino acids respectively, within the GvpC protein. At that site they may be less readily accessible to the intracellular degradation processes. Nevertheless they are immune visible and evoke specific antibody responses.

Gas vesicles are particulate moieties and when phagocytosed can impact various cell functions, including cytokine production. In turn, both individually and in combination, cytokine levels significantly affect the nature and level of immune responses and response polarization in terms of antibody (Th2), and cell mediated immunity (Th1) [21,22]. *In vivo*, cytokines impact immune related processes and certainly specific roles for different cytokines have been identified and extensively investigated. Studies of HIV for example, have demonstrated cytokine correlations with protection from infection, prediction of disease progression and impact on immune response polarization [23-26]. Based on the potential for an insert specific effect, the present *in vitro *studies assessed the r-GV immunogens for their possible impact on selected cytokines, quantified by testing the media of monolayers treated with r-GV^Tat.Rev and Nef1^, vs. wt-GV, or left untreated. Although the SIV specific peptide inserts constitute a very small segment in the recombinant GvpC protein, nevertheless *in vitro *studies showed there were detectible, insert specific differences in the cytokine patterns and the temporal profiles of their changing concentrations (Table 2). Given the known effects of cytokines IL-18, IL-10 and IL-12, and data in the literature relating to Tat, Ref and Nef1, the present findings show that despite the preponderance of GvpC specific protein, even very small segments of these pathogen proteins could play specific and significant roles *in vivo *by influencing the local milieu [27-29]. Although *in vitro *effects can not fully reflect *in vivo *process or responses, they nevertheless can indicate the potential to exert an effect. Analogous consequences *in vivo *could alter the local milieu and thereby alter the Th1/Th2 responses equation. In turn, this might affect vaccine efficacy and certainly adds to a characterization of this immunization vehicle. Thus similar examinations would be relevant in the broader context of vaccine development, and of characterizing pathogen impact on tissues or systems. Known examples are inhibitory effects of IL-10 on HIV-1 production in monocytes/macrophages resulting from IL-10 stimulated inhibition of cytokine syntheses e.g. tumor necrosis factor alpha (TNF-α) and IL-6. In the context of HIV-1, this alteration can up-regulate pathogen expression in the infected cells [30]. Additionally, studies by others also have described IL-10 inhibitory effects on cytokine synthesis [31-33], and on pathogen (HIV) replication in monocytes [30,34].

The inherent physical stability of the gas vesicle structure, along with its resistance to chemical dissociation or proteolytic cleavage [12,35,36] and its particulate structure, likely play a role in the natural slow release of the incorporated epitope containing fusion proteins and could enhance stimulation of the immune system. Ultimately however, the full degradation of the particulate r-GV immunogen is important. Their long term presence potentially could elicit chronic, local inflammatory responses that can characterize degradation resistant particles. The *in vitro *immunostaining and the Western blot findings presented here were aimed at assessing intracellular GV breakdown. The results indicate that degradation of these naturally stable immunogenic particles does occur and demonstrates the sizes of degradation products. In this context, it is noteworthy that even long term, oral ingestion of gas vesicles on a regular basis [37] has provided no evidence that gas vesicles exhibit any detectible short or long term toxicity. Thus per-oral usage of r-GV also could be an option.

Micrographs from the immunofluorescence staining allow the tracking of r-GV intracellular fate after the application to J774A.1 cells monolayers *in vitro*. These studies demonstrated that as expected, r-GV are phagocytosed. Microscope focusing indicated r-GV associated with and/or were within J774A.1 macrophages at 12 h post application and clearly internalized at later times (see Figures 2 and 3). Results from the subsequent immunostain monitoring of both the gas vesicle protein GvpC, and the SIV specific inserts confirmed their slow, progressive disappearance over time. An intracellular accumulation/coalescence of individually internalized r-GV particles clearly takes place and is evident in the micrographs of 12 h post application monolayers. Additionally, samples from various times (12-120 h) following removal and rinsing off of the applied r-GV^Tat, Rev or Nef1 ^were immunostained using either anti- SHIV or anti-GvpC antibody (Figure 3 and 4) and examined. These clearly show a decrease in detectible immunostained material during this time course. The results from this group of studies therefore provides critical information in terms of potential r-GV utility as a delivery vehicle since it indicates that *in vivo *there should be an ongoing but distinctly finite host cell perturbation following cellular uptake. This likely would impact immune stimulation, potentially by processes including the stimulation of cytokine production detected and quantified above. Importantly the present studies tracking degradation indicated that over time *in vitro*, the recombinant particles did undergo degradation in the individual cells. This was evidenced by the immunomicroscopy data for samples (Figures 3 and 4) and was verified in more detail by the Western blot assessments which tested lysates of r-GV treated monolayer at 96 and 120 h post r-GV applications (Figure 5).

Since recombinant inserts are expressed in gas vesicle protein 'C', studies assessed breakdown of this protein species and also of the specific expressed SIVsm inserts. In SDS-PAGE gels isolated, intact GvpC would appear as a single, ~60 kDa protein species [9]. The Western blot analysis using anti-GvpC showed clearly that wt-GV and the r-GV^Tat, Rev and Nef1 ^recombinants all exhibited multiple molecular weight species at both 96 and the 120 h times after application to monolayer cells. This is the predicted result when proteins that become internalized are broken down and is consistent with the immunostaining detected in Figures 3 and 4. It also would be expected that the band patterns in SDS-PAGE gels, and the density of Western blot immunostained bands would change as a function of time and this is the case (Figure 5, left hand panels). Thus, by 120 h, there are crisp GvpC positive bands of lower molecular weight species (ex. somewhat greater than 18 kDa and less than 14 kDa), as well as higher molecular weight species which are now more lightly stained by the anti-GvpC antibody. All of these bands are reactive with the anti-GvpC antibody, and since in SDS-PAGE gels, GvpC that is un-degraded behaves as a 60 kDa moiety [9]; these clearly are degradation products from the GvpC protein. They exhibit discrete, decreasing molecular weights consistent with degradation that is ongoing over time. Similarly the companion Western blots probed with anti-SHIV specific antibody demonstrated breakdown of the SIVsm derived inserts and showed the disappearance of bands over time (Figure 5, right hand upper and lower panels). In addition to the detailed findings, the results overall support the conclusion that intracellular r-GV are susceptible to degradation and therefore can be cleared from cells that may phagocytose them. Furthermore, unlike the effects on cytokine concentrations, inserts in r-GV^Tat, Rev or Nef1 ^do not appear to significantly alter the r-GV time course of degradation in an insert dependant way.

## Conclusions

The studies presented here provide an initial examination of recombinant gas vesicles (r-GV) in terms of predominant antibody isotype elicited *in vivo*, particle impact on selected cytokines elicited following uptake by monolayer cells *in vitro *and r-GV internalization and intracellular degradation during *in vitro *culture. The murine immunizations showed that despite the relatively small size of the expressed recombinant insert, like other haptens SIVsm derived inserts incorporated into the GvpC "carrier" protein on gas vesicles surfaces, are clearly visible and can initiate immune responses. Additionally, the level of murine humoral responses observed likely would reflect effects from gas vesicle elicited cytokines and the natural slow but progressive release of antigen over time.

The complexity, functions and detailed interactions of cytokine and their network are well outside the scope of these studies; however *in vitro *effects of cell perturbation by gas vesicles and their SIV derived inserts could establish a profile of cell responses relevant in the context of an antigen delivery system. Screenings for possible insert specific effects examined IL-10, IL-12 and IL-18 which have been implicated as protective during infection by intracellular pathogens [21,38-41]. Therefore studies quantified these three to obtain a profile of insert specific effects. The assay results showed that despite the small size of the SIV inserts, changes are evoked that differ quantitatively and temporally depending on the recombinant r-GV applied. Thus each had unique, signature effects when phagocytosed by cells and therefore would exert a different but characteristic impact when used as an immunogen. These findings indicate that cytokine analyses that targeted examining the effect of individual peptide immunizing agents could be highly relevant as an additional parameter that aids in characterizing the epitope(s) of those agents.

The *in vitro *time course examining r-GV uptake and fate used immunostaining with rabbit anti-GvpC and monkey anti-SHIV antibodies to detect and assess a time course for intracellular breakdown of phagocytosed organelles. The distribution of fluorescence labelling demonstrated the progressive loss over time of protein specific immunostaining. As a larger protein species there was an inherently slower clearance of the gas vesicle protein, GvpC vs. the viral inserts which are small peptides. In terms of *in vivo *host immune responses, slower degradation would extend the period of cell perturbation. In turn this could impact cells and their processes - antibody production, specific antibody isotype(s) generated, and the specific cytokine profiles elicited.

The complexity, functions and detailed interactions of cytokines and their network were outside the scope of these studies but the *in vitro *effects of cell perturbation by gas vesicles indicated screening for possible insert specific effects on IL-10, IL-12 and IL-18 would be a highly relevant parameter in defining the SIV epitopes of interest here [21,37-40]. These cytokines therefore were quantified to obtain a profile of insert specific effects. The results showed r-GV^Tat, Rev and Nef1 ^each evoke changes that are quantitatively and temporally different. Therefore each likely would have unique, signature effects when phagocytosed by cells and thus would exert different but characteristic perturbations. The impact of Th1/Th2 balance on immune responses to antigen perturbation is crucial: Th1 responses supports inflammation and T and macrophage cell activation, while Th2 actives primarily B cells, resulting in antigen specific antibody. The cytokine quantifications were carried out as described using the *in vitro *cell culture system and there were notable temporal and quantitative differences. Clearly responses generated *in vivo *vs. *in vitro *can differ significantly as the former is a far more complex environment. Thus, in the absence of *in vivo *data there is no valid basis to speculate about the effect of this mode of antigen presentation *in vivo*. However, the findings suggest that cytokine analyses to establish the effect(s) of individual peptide immunizing agents delivered by r-GV could be generally useful as an additional parameter characterizing epitopes that are being selected for vaccine usage.

Cell uptake of r-GV^Tat, Rev or Nef1 ^results in characteristic; insert specific changes in the three cytokines assessed. The precise mechanisms of immune response regulation *in vivo *are highly complex. However, for particulate antigens, such as those tested here, internalization and degradation by dendritic cells or macrophages would be a critical initial step in the process. The *in vitro *findings indicate that the highly stable gas vesicle particle is readily and rapidly internalized by macrophages such as the J774A.1 cell line used here. The *in vitro *time course demonstrating the intracellular breakdown used rabbit anti-GvpC and monkey anti-SHIV antibodies. It demonstrated the progressive loss of protein specific immunostaining over time and as expected, the larger Nef1 insert was degraded more slowly than the Tat and Rev components. The later stages of this process also were examined by Western blot. As expected, these clearly showed the array of molecular weight species formed during intracellular breakdown. It demonstrated the progression of changes and verified that at the 120 h time, breakdown remained incomplete and thus would continue to perturb the cell. As a larger protein species the inherent slower clearance GvpC vs. the viral insert likely reflects primarily the larger size of the GvpC component. However, in terms of host immune responses, GvpC's slower disappearance, an indicator of degradation, likely results in an extended period of cell perturbation. *In vivo *perturbation can impact processes such as stimulation of antibody production, the specific antibody isotype(s) that ultimately is formed, and the changing profile of cytokines produced.

Finally, the results overall support the conclusion that although the SIV inserts tested here differ in amino acid sequences, expressed peptide size and *in vivo *function during infection, both the Western blot data, and the fluorescent microscopy studies show the inserts do not significantly alter the time course for r-GV degradation. Interestingly, intact Rev and also Tat proteins would normally track into the nucleus during *in vivo *infection by HIV [42,43]. The immunostained 120 h micrographs, demonstrate that numerous cell nuclei specifically reacted with the anti-SHIV antibody, some even exhibiting a green "glow". Thus data from the r-GV^SIV ^treated monolayer cultures suggest that in this *in vitro *system, the Rev peptides in particular, but also Tat can enter the nuclear compartment of J774A cells. Potentially therefore, an inherent characteristic, amino acid sequence remains fully functional when expressed in GvpC-SIV protein chimeras and effectively targets the nucleus.

## Methods

### Isotype determinations

Isotype identification used sera elicited by initial immunizations with r-GV followed at intervals by boosters of 50 μg r-GV/mouse using the regimen presented in Table 1 and assessed for anti-r-GV titer by ELISA [8]. These sera, designated based on the portions of the viral gene segment they expressed (e.g. *tat*-150 bp, *rev*-243 bp and *nef1*-642 bp) were stored at 4°C and further examined here to determine the predominant isotype of the antibody that specifically recognizes the SIVsm-sequences expressed by the different r-GV populations. Antibody to gas vesicle proteins present in anti-SIVsm-Tat, -Rev, or -Nef1 mouse sera was adsorbed by sequential incubation with wild type gas vesicles (wt-GV) and the absorbed sera then titered using standard ELISA methods and the appropriate recombinant gas vesicle preparations (r-GV^Tat, Rev, or Nef1^) as the antigen [8]. That optimum antibody dilution then was isotyped using reagents from SouthernBiotech (1070-04, 1080-04, 1021-04; SouthernBiotech, Birmingham, AL) which provide the necessary isotype specific antibody. The manufacturer's protocol was followed and except as noted, incubation was carried out for 1 hour (h) at room temperature (RT). Gas vesicles function as flotation devices for *Halobacteria *and were routinely collapsed (deflated) by brief centrifugation using a microfuge as described previously [8]. Collapsed gas vesicles will settle onto and associate with wells of ELISA plates, and likewise will associate with monolayer cell surfaces allowing subsequent phagocytic uptake by these macrophages. Briefly, ELISA wells were coated with the appropriate collapsed gas vesicles diluted to 1 μg/100 μl in PBS, and subsequently blocked with 1% BSA/PBS, then incubated with mouse antisera appropriately diluted in 0.1% BSA/PBS. Control wells in the plates were also coated using the same collapsed gas vesicle preparations but received 0.1% BSA/PBS containing no primary antibody. After rinsing 4×, each well received 100 μl of a 1:1,000 dilution of pre-conjugated isotype specific antibody and plates were again incubated for 1 h at RT. Wells were then rinsed 5× with 0.1% BSA/PBS, and incubated at 37°C with 100 μl of pNPP substrate solution. Absorbance readings at 405 nm were taken at 10, 20, 30 and 60 min, using a VersaMax ELISA Reader (Molecular Devices, Palo Alto, CA).

### Cell Culture and Application of Isolated Gas Vesicles

Murine macrophage J774A.1 cells were grown to 75-80% confluence on 12-mm glass cover slips (Fisher Scientific, Pittsburg, PA) in 12 well plates (BD Falcon, Franklin Lakes, NJ) using the Richter's Improved Minimum Essential Medium with Insulin (IMEMZO, Irvine Scientific, Santa Ana, CA) containing 10% Fetal Bovine Serum (FBS, Atlanta Biologicals, Norcross, GA) at 37°C and 5% CO_2_. At ~ 80% confluence the media was removed and the cells washed 3× with fresh IMEMZO, 500 μl/well. For cultures used in the cytokine assays wild type gas vesicles (from *Halobacterium *NRC-1) as well as recombinant gas vesicles, previously purified, dialyzed and collapsed by microcentrifugation [8] were diluted in sterile serum free media (SFM) (QBSF^®^55, Sigma-Aldrich, Inc., Saint Louis, MO; Q3878) and 400 μl/well sterilely added to the appropriate wells to give a final concentration of 500 μg/ml. Wells serving as controls for the effect on cytokine levels of *in vitro *cell culturing per se, received SFM containing NRC-1 gas vesicles. For studies of gas vesicle degradation, SFM was used throughout. These monolayers also received 400 μl/well of the 500 μg/ml isolated gas vesicle stock, delivered in media. For the degradation studies the control wells received SFM without gas vesicles and for both studies, plates were incubated at 37°C with 5% CO_2 _for 2.5-3 h. After incubation, the recombinant gas vesicles containing media was removed from the wells and monolayers were rinsed 1× with PBS. Each plate received 300 μl/well of SFM and the plates again incubated using the conditions noted above. For cytokine assays, culture supernatants were harvested at time points between 12-48 h post gas vesicle application, passed through a 0.2 μm syringe filter (Gelman Sciences, Ann Arbor, MI) to remove any cell debris, and then individually stored at - 20°C until assayed for cytokines. For the gas vesicle degradation studies, coverslips with monolayers were harvested at different times between 12 and 120 h post application, rinsed 3× with PBS, fixed using the cold 70% methanol (500 μl/well), and stored in sealed culture plates at - 20°C for subsequent immunostaining as described below under intracellular degradation.

### Murine cytokine measurement by ELISA

Assays assessed three selected murine cytokines, potentially present in the tissue culture media of murine J774A.1 macrophage monolayers, harvested and stored at - 20°C as described above. Assessments used the following commercial kits: Mouse IL-18 ELISA Kit (#7625; Medical & Biological Laboratories Co., Ltd. Naka-ku Nagoya, Japan), Endogen^® ^Mouse IL-12 ELISA Kit EMIL12, and the Endogen^® ^Mouse IL-10 ELISA Kit, EMIL 10 (Pierce Biotechnology, Inc., Rockford, IL). The IL-18 *in vitro *ELISA assay kit uses a sandwich format. The two Endogen^® ^kits are direct assays to measure the quantity of biologically active mouse IL-12 (p70) and IL-10 in culture supernatants, after it has bound to test plates. Cytokine assay of the standards and experimental samples were carried out in duplicate. Using the computed average, ± standard deviation, standard curves were generated and the average of duplicates for each experimental sample used to determine picogram quantities of each cytokine in each sample at each different time point.

Briefly, the IL-18 ELISA Kit is a capture assay system using wells that were commercially coated with an anti-IL-18 monoclonal antibody. 100 μl of diluted IL-18 standard (1,000, 400, 160, 64, 25.6 and 0 pg/ml) or sterile culture supernatant in duplicate was incubated in the wells for 1 h at RT, rinsed 4× with washing solution provided in the kit. The IL-18 "captured" from the culture media then was detected with a second monoclonal antibody pre-conjugated with horseradish peroxidase (HRP). After incubation for 1 h at RT, wells were rinsed 4× with washing solution. Wells were filled with the chromogen tetramethylbenzidine/hydrogen peroxide (TMB), supplied with the kit and incubated 30 min at RT. An acid solution (0.5 M H_2_SO_4_) then was added to each well to terminate the enzyme reaction and to stabilize the developed color. The optical density (OD) of each well is then measured at 450 nm using the VersaMax microplate reader. The concentrations of mouse IL-18 were determined from the standard curve generated based on the absorbance of dilution series of the IL-18 reference standard provided with the Kit. The total concentration of mouse IL-18 for sample then is determined by multiplying the value read from the standard curve by the dilution factor for the sample.

The Endogen^® ^mouse IL-12 and IL-10 assay kits included interleukin standards for cytokine quantification and were used as specified by the manufacturer. Prepared standards and samples were added to the anti-Mouse IL-12 (p70) pre-coated wells in duplicate. The plate was covered and incubated at RT for 2 h. Without washing the plate, prepared biotinylated antibody reagent was added to each well and incubated for 1 h. After washing the plate 3× with wash buffer, ready to use streptavidin-HRP solution was added and the plate was incubated at RT for 30 minutes. Following another washing, premixed TMB Substrate solution was added and the plate was incubated in the dark for 30 minutes. The reaction blocked by adding stop solution and the absorbance measured on the plate reader at 450 nm. The results were calculated using curve fitting statistical software of the VersaMax instrument. For IL-10 detection, the procedures were similar with the following exceptions: standards and sample were incubated for 3 h at RT and following this incubation, plates were rinsed 3× with wash buffer before the biotinylated anti-IL-10 antibody was added. As with the IL-12 kit, the reaction was terminated with stop solution, the absorbance measured on the VersaMax plate reader at 450 nm and the results calculated using the VersaMax curve fitting statistical software.

### Intracellular degradation of gas vesicles

Assessment of phagocytosed gas vesicle intracellular fate used the fixed, stored J774A.1 monolayers and application of isolated, collapsed wild type or recombinant gas vesicles described above in the cell culture methods. Samples fixed at the 12, 24, 48, 72, 96 and 120 h post gas vesicle application times were immunostained to identify GvpC or the SIVsm derived moieties: Tat, Rev and Nef1. After rinsing these monolayers with PBS, specific detection used either purified rabbit anti-GvpC antibody (concentration 0.335 μg/μl) or plasma from an SHIV-infected macaque (SHIV variant 89.6PD from animal number R94085; a gift from Dr. D. Pauza, Institute of Human Virology, Baltimore, MD). These antibodies were diluted 1:200 in PBS and 200 μl/well added to the appropriate monolayers. Plates were then incubated at 37°C for 1 h. After rinsing 3× with PBS, cells were incubated with a 1:50 dilution of FITC (fluorescein isothiocyanate) conjugated goat anti-rabbit secondary antibody (Jackson Immuno Research, South Windham, ME) or with FITC conjugated rabbit anti-monkey IgG (F3893; Sigma Chemical Co.; St. Louis, MO). Following incubation at RT for 45 minutes, coverslips were rinsed with PBS, then mounted on microscope slides using Vectashield (H-1200; Vector Laboratories, Burlingame CA) and sealed. Subsequently they were examined and digitally documented using an Olympus BX41 microscope and Olympus digital camera C-5050.

### J774A.1 Cell lysate assessment by Western blot

Gas vesicle breakdown/intracellular protein degradation also was assessed by Western Blot. Briefly, monolayers of J774A.1 cells grown in 24 well plates were then incubated with r-GV^Tat, Rev or Nef1 ^preparations, or with wt-GV as described above. After removal of the GV and addition of fresh media, cells were further incubated for 48, 96 or 120 h. Cells were harvested from the wells at those time points, lysed in a 5× SDS-PAGE sample buffer (Pierce-Endrogen, Rockford, IL), then heated in a boiling water bath for 5 min. Aliquots of 20 μl were loaded into wells of a pre-cast 4-12% NuPAGE^® ^Novex Bis-Tris Gels (Invitrogen, Carlsbad, CA) and following electrophoresis, usually 45-50 min, the separated proteins were transferred to a PVDF membrane (Millipore, Bedford, MA) using a BioRad transfer apparatus (BioRad, Hercules, PA) following the manufacturer's instructions. SeeBlue PreStained Standard (Invitrogen, Carlsbad, CA) was used to assess the molecular weight of the proteins. Subsequently, membranes were blocked with 5% non-fat dry milk (NFDM) in PBS. This and all other incubations were carried out at RT on a shaking platform. After blocking for 2.5 h, membranes were rinsed three times with 1% BSA/PBS. To identify the GvpC protein, half of each blot was incubated with 0.020 μg/μl of rabbit anti-GvpC antibody diluted in 1% BSA/PBS. To identify SIVsm peptides expressed in the recombinant gas vesicles, the other membrane half was incubated with anti-SHIV macaque plasma described above, diluted 1:200 in 1% BSA/PBS. Following 1.5-2 h incubation with appropriate anti-sera, membranes were rinsed three times in 1% BSA/PBS. Specifically bound antibody was detected by incubating membranes with either Alkaline Phosphatase (AP) conjugated goat anti-rabbit IgG (H+L; Jackson Immuno Research, West Grove, PA) or with alkaline phosphatase (AP) conjugated rabbit anti-monkey antibody (F3893; Sigma-Aldrich, St. Louis, MO). Each secondary antibody conjugate was diluted 1:1,000 in 1% BSA/PBS and incubation continued for 1 h. Membranes were rinsed three times with 1% BSA/PBS and twice with dH_2_O, and then incubated with the alkaline phosphatase (AP) membrane substrate, Sigma Fast (Sigma-Aldrich, St. Louis MO); protein bands developed within 5 min of substrate addition. Subsequently blots were rinsed in distilled water; air dried at RT and archived as scanned images.

## Abbreviations

wt-GV: wild type gas vesicles; r-GV: recombinant gas vesicles; Gvp: gas vesicle protein; SIV: Simian Immunodeficiency Virus; SIVsm: Simian Immunodeficiency Virus infecting the sooty mangabeys; SHIV: Simian-Human Immunodeficiency Virus; bp: base pair; BSA: Bovine Serum Albumin; OD: optical density; PBS: Phosphate-Buffered Saline; PCR: polymerase chain reaction; RT: room temperature; SFM: serum free media; HRP: horseradish peroxidase; TMB: tetramethylbenzidine/hydrogen peroxide; FITC: fluorescein isothiocyanate; NFDM: non-fat dry milk; AP: Alkaline Phosphatase; Ig: Immunoglobulins; AIDS: acquired immunodeficiency syndrome; PVDF: Polyvinylidene fluoride (membrane); FBS: Fetal Bovine Serum; pNPP: para-nitrophenyl phosphate; Tat: a 50 amino acids peptide segment encoded by the *tat *gene; Rev: a peptide segment containing 81 amino acids; Nef1: a peptide segment encompassing amino acids 1-214, of the protein encoded by the *nef *gene.

## Authors' contributions

This work was a portion of doctoral studies carried out in partial fulfilment of the requirements for a Ph.D. in Microbiology at the University of Massachusetts (MS). Both authors contributed substantially to the work and manuscript presented here. As senior author and PI of the study, ESS developed the plan, supervised the work, and finalized the manuscript. Both authors read and approved the final manuscript.
